# Draft Genome Sequence of a Novel *Luteimonas* sp. Strain from Coral Mucus, Hawai‘i

**DOI:** 10.1128/genomeA.01228-16

**Published:** 2016-11-03

**Authors:** Xuehua Wan, James M. Miller, Sonia J. Rowley, Shaobin Hou, Stuart P. Donachie

**Affiliations:** a Advanced Studies in Genomics, Proteomics and Bioinformatics, University of Hawai‘i at Mānoa, Honolulu, Hawai‘i, USA; b Department of Microbiology, University of Hawai‘i at Mānoa, Honolulu, Hawai‘i, USA; c Department of Geology and Geophysics, University of Hawai‘i at Mānoa, Honolulu, Hawai‘i, USA

## Abstract

*Luteimonas* sp. strain JM171 was cultivated from mucus collected around the coral *Porites lobata*. The JM171 draft genome of 2,992,353 bp contains 2,672 protein-coding open reading frames, 45 tRNA coding regions, and encodes a putative globin-coupled diguanylate cyclase, *Jm*GReg.

## GENOME ANNOUNCEMENT

We cultivated the yellow-pigmented strain JM171 during an investigation of culturable bacteria in mucus produced by the coral *Porites lobata* in Kewalo Basin, O’ahu, Hawai’i. The nearest validly described neighbors on the basis of a BLAST comparison of the JM171 16S rRNA gene nucleotide sequence are *Luteimonas* species, with identities ranging from 95 to 96% (1–12). However, an EzTaxon alignment suggests the nearest neighbor is *Luteimonas dalianensis* OB44-3^T^ (98.4%), a species not on the List of Prokaryotes with Standing in Nomenclature (http://www.bacterio.net/luteimonas.html) (13–15). The 11 recognized *Luteimonas* species were isolated from a range of aquatic habitats, largely marine, and soil or plant surfaces, and are all yellow-pigmented (2–12). The genus is part of the *Xanthomonadaceae* in the *Gammaproteobacteria*.

We sequenced the genome of *Luteimonas* sp. strain JM171 to determine if the strain has pathways that may be involved in establishing or maintaining an association with *P. lobata*, such as to provide compounds that are beneficial or detrimental to a coral host, or expression of molecules that deter settling or grazing by other microbes or animals.

Genomic DNA isolated using the Wizard genomic DNA purification kit (Promega, USA) was used to prepare a Roche 454 GS FLX+ Titanium rapid library and an 8-kb-span paired-end library for genome sequencing, according to Roche protocols. A total of 103.4 Mb of shotgun sequences were generated. The initial assembly comprised 38 contigs covering ~3 Mb. To build the scaffold and derive orientation information for the contigs, we produced 7.8 Mb of paired-end sequences. Newbler 2.8 built a single scaffold containing 23 contigs covering an estimated 2,993,395 bp. Among the assembled bases, 99.98% have a base accuracy quality above a Phred quality score of Q40. The average read coverage is ~37×. Most gaps were closed as previously described (16). The final scaffold totals 2,992,353 bp.

The genome was annotated in the NCBI Prokaryotic Genome Annotation Pipeline (PGAP) (17) and the Rapid Annotation Using Subsystem Technology (RAST) server (18, 19). PGAP identified 2,672 protein-coding genes, 45 tRNA-coding regions, and three rRNA-coding regions. RAST identified 2,750 protein-coding open reading frames and 44 tRNA-coding regions. We manually predicted one putative globin-coupled diguanylate cyclase, *Jm*GReg (BGP89_03560), of which the primary sequence presents homologous domains of *Bpe*GReg and *Ec*GReg/DosC, from *Bordetella pertussis* and *Escherichia coli* respectively, that sense oxygen to regulate the production of intracellular second messenger c-di-GMP (20, 21). The G+C content of the JM171 genome is 69.05 mol%. The strain appears unlikely to be a strain of *L. dalianensis*, in which the G+C content is 64.6 mol% (15).

### Accession number(s).

This whole-genome shotgun project has been deposited at DDBJ/EMBL/GenBank under accession number CP017074. The version described in this paper is the first version.
